# Recent Advances in Computer-Assisted Algorithms for Cell Subtype Identification of Cytometry Data

**DOI:** 10.3389/fcell.2020.00234

**Published:** 2020-04-28

**Authors:** Peng Liu, Silvia Liu, Yusi Fang, Xiangning Xue, Jian Zou, George Tseng, Liza Konnikova

**Affiliations:** ^1^Department of Biostatistics, University of Pittsburgh, Pittsburgh, PA, United States; ^2^Department of Pathology, University of Pittsburgh, Pittsburgh, PA, United States; ^3^Department of Pediatrics, University of Pittsburgh, Pittsburgh, PA, United States; ^4^Department of Immunology, University of Pittsburgh, Pittsburgh, PA, United States; ^5^Department of Developmental Biology, University of Pittsburgh, Pittsburgh, PA, United States

**Keywords:** CyTOF, manual gating, cell type identification, clustering, auto-gating, visualization

## Abstract

The progress in the field of high-dimensional cytometry has greatly increased the number of markers that can be simultaneously analyzed producing datasets with large numbers of parameters. Traditional biaxial manual gating might not be optimal for such datasets. To overcome this, a large number of automated tools have been developed to aid with cellular clustering of multi-dimensional datasets. Here were review two large categories of such tools; unsupervised and supervised clustering tools. After a thorough review of the popularity and use of each of the available unsupervised clustering tools, we focus on the top six tools to discuss their advantages and limitations. Furthermore, we employ a publicly available dataset to directly compare the usability, speed, and relative effectiveness of the available unsupervised and supervised tools. Finally, we discuss the current challenges for existing methods and future direction for the new generation of cell type identification approaches.

## Introduction

Cytometry is a field of measuring molecular and physical characteristics of individual cells used both in clinical practice and research settings that has allowed for significant advancements in medicine and biology. This can be used for studying cells in suspension, the focus of this review with various methods described below, or adherent cells by image cytometry, reviewed elsewhere. For several decades, flow cytometry has enabled simultaneous identification of multiple features or antigens found on the surface or inside individual cells at a single cell resolution. This technique relies on the detection of fluorescence emitted by fluorophore conjugated antibodies that emit fluorescence at particular wavelength upon excitation by specific lasers (Bendall et al., 2012; Comi et al., 2017). It can be applied to any cellular suspension both for cellular analysis and for cell sorting to isolate specific groups of cells using panels of antibodies. The number of fluorophores that can be combined and simultaneously detected is limited by the number of lasers available and the spectral overlay of each fluorophore used. Additionally, the spectral overlay between various fluorophores creates an overlap between them that requires compensation of the data generated to ensure specificity and limit the interaction between the fluorophores, a process usually accomplished by using single-color controls (beads or cells that are stained for one fluorophore at a time) (Doerr, 2011). Moreover, to eliminate the background contribution of cellular autofluorescence, unstained controls must be included in the experiment. Routinely, panels have consisted of 8–10 antibodies.

With advancement of flow cytometers such as implementation of multiple lasers and increase in available reagents, ∼20–30 antigens can be reliably measured (Verschoor et al., 2015). Additionally, recent advancement in the cytometry field such as spectral cytometry (Aurora, Cytek) and mass cytometry (CyTOF, Fluidigm) have further extended these capabilities with ability to measure 30–60 individual markers simultaneously. Spectral cytometry relies on simultaneous detection of the full emission spectrum of each fluorochrome used across all lasers instead of just the peak emission that is detected in standard flow cytometry (Schmutz et al., 2016). This allows one to combine fluorochromes with similar peak emission but distinct full emission signatures into the same panel greatly expanding upon the flow cytometry capabilities (Ferrer-Font et al., 2019). In CyTOF, instead of fluorophores, the markers of interest are labeled with stable heavy metal isotopes that are rarely present in live cells and detected by time of flight mass spectroscopy. A detailed review and comparison of these platforms is beyond the scope of this review, but they have been extensively reviewed in many other publications (Maaten and Hinton, 2008; Schmutz et al., 2016; Hartmann and Bendall, 2019; Zielinski, 2019).

The focus of this review, on the other hand, is to evaluate the tools that are available for cellular populations clustering and identification using multi-parameter cytometry data. Ever since its development in 1984 by the Society for Advancement of Cytometry (ISAC), flow cytometry data and subsequently spectral and mass cytometry data, are all stored by convention in Flow Cytometry Standard (FCS) format files (Murphy and Chused, 1984). These files contain textual metadata describing the experiment combined with binary data of the results. The data are stored as an array or a matrix where each row is an event or an individual cell and each column is a value that corresponds to the magnitude of the signal in a particular “channel.” For flow cytometry data, these channels correspond to either the fluorophores used in the experiment or the scatter channels that correspond to the light scattered by the individual cells passing through a flow cell. In mass cytometry data, channels correspond to the heavy metals. Additionally, for flow cytometry data, a compensation matrix, or a correction factor, that adjusts for the spillover of a primary channel into other channels obtained from single-color controls mentioned above, must be applied to the data before analysis (Bagwell and Adams, 1993; Roederer, 2001).

There are several approaches to analyze cytometry data. Traditionally, this has been accomplished by manual gating, a sequential selection of specific parameters represented by the channels in the FCS files, to identify the populations of interest. This process is known as hierarchical sequential gating strategies (Bendall et al., 2012; Verschoor et al., 2015) and is usually accomplished by plotting bi-axial dot plots that compare two parameters (two channels) at a time and manually drawing “gates” representing the positive or negative population for the particular parameter combination. Manual gating was the earliest method used to define known cell populations from cytometry data, e.g., CD45^+^ CD3^+^ for T cells, and continues to be widely applied. Various tools are available to aid with manual gating that include commercially available platforms or those available through R packages. However, relying solely on manual gating for interpretation of high-dimensional cytometry data with a large number of parameters has its limitations. It is laborious, time-consuming and depends on the end user’s fundamental understanding of what markers define specific cellular populations. Moreover, it is subjected to human bias both during the manual gating process when the end user identifies where to draw positive and negative gates to define cellular populations as well as on preconceived notions of what antibodies mark particular cellular populations. Furthermore, biaxial plots are unable to capture the increased complexity of cellular populations afforded by high dimensional panels. Therefore, novel computational approaches are needed to capture the complexity allowed by the higher dimensional data acquired through mass and spectral cytometry and higher dimensional flow cytometry.

As such, a number of automated tools have been developed including spanning-tree progression analysis of density-normalized events (SPADE), Phenograph and Self-Organizing Map (FlowSOM) among many others, that organize individual cells with similar marker expression into clusters or categories (Qiu et al., 2011; Levine et al., 2015; Van Gassen et al., 2015). These clusters can then be further annotated to provide biological relevance based on their markers’ expression. For example, a particular cluster might have high values for CD45, CD3, CD4, CD45RA, and CCR7 and would therefore represent naïve helper T cells or a different cluster might be low for CD45 and high for EPCAM representing epithelial cells. As such, instead of sequentially defining populations of interest as done in manual gating, in automated clustering, the populations are already identified by the algorithm and the end user assigns biological relevance to each cluster by their overall marker expression.

**FIGURE 1:**
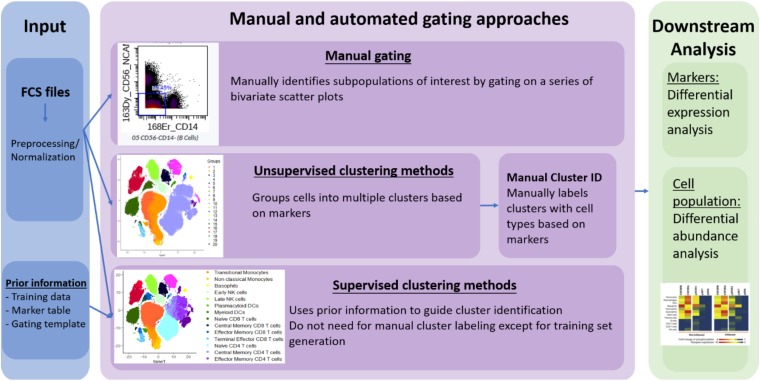
Overview of manual gating, unsupervised and supervised clustering tools for high-dimensional cytometry data analysis.

However, no standard nomenclature has been developed to apply to these tools. In this review, we propose to categorize the available computer-assisted automated cell clustering algorithms into three major categories: (1) unsupervised clustering methods, (2) supervised clustering methods, and (3) trajectory inference (TI) methods. Unsupervised clustering tools group cells into categories based on their marker expression using computational machine learning algorithms without a requirement for any prior knowledge while supervised clustering methods rely on prior knowledge or supplemental information for the tools to properly cluster the cells or annotate the cellular clusters generated. TI algorithms, on the other hand, are used to establish a relationship or a trajectory between the cellular groups via an unsupervised computational method. As there are several published comprehensive TI reviews (Cannoodt et al., 2016; Saelens et al., 2019) that benchmark the available algorithms and provide general guidelines for their applications, this review will focus exclusively on unsupervised and supervised clustering algorithms. In Table 1 and Figure 1 we provide a big picture comparison between (1) unsupervised clustering tools, (2) supervised clustering tools and manually gated data.

**TABLE 1:** Comparison of manual gating, unsupervised and supervised clustering methods.

	**Manual gating**	**Unsupervised clustering methods**	**Supervised clustering methods**
Ease of use	Easy and straight forward for biologist	Tool dependent, generally easy to apply. See Table 3	Tool dependent, generally requires more steps than unsupervised clustering methods
Reproducibility	Reproducible between data for same user	Majority of the tools allow for setting a “seed” enabaling the reproducibility of the results. See Table 3	Variable (tool dependent)
Time cost	Experience and sample size dependent	Tool dependent, see Table 3	Tool dependent, generaly high. See Table 4
Flexibility	High, depends on user manual setting	Moderate, users can only adjust some parameters	Low
Novel subpopulation detection	Yes	Yes (tool dependent)	No (can only detect previously defined clusters)
Subpopulation/cluster identification	Manual (based on gating strategy)	Manual (based on cluster marker expression)	Automated (based on training set)
# of subpopulations/clusters	Experiment dependent	Variable (some allow users input; some automatically optimize #) See Table 3	Fixed (based on training set)
Prior knowledge requirement	Gating Experience, Marker expression for cellular identification	None for clustering; knowledge of marker expression for cluster identification	Training dataset or marker matrix, familiarity with bioinformatics

### Overview of Clustering Tools Reviews

Several groups have reviewed many of the such computational tools (Chester and Maecker, 2015; Mair et al., 2016; Saeys et al., 2016; Weber and Robinson, 2016; Nowicka et al., 2017; Kimball et al., 2018; Mair, 2019; Todorov and Saeys, 2019; Zielinski, 2019). These publications are summarized in Table 2. Most of these reviews have focused on a selected subgroup of 5–10 tools from those available, to illustrate how automated clustering and visualization methods can facilitate cellular population’s identification. Many of these tools can be applied to any high dimensional cytometry data. Of the reviews summarized in Table 2, the two by Nowicka et al. (2017) and Kimball et al. (2018) focus more on applying the computational tools to CyTOF data, while Saeys et al. (2016) focus on flow cytometry data and Weber and Robinson (2016) apply the tools to both flow and mass cytometry data.

**TABLE 2:** Overview of reviews of clustering tools.

**Title**	**Citation (Author, year)**	**Focus of the paper (datasets discussed)**	**Visualization tools**	**Clustering tools (#: tool names)**	**Trajectory inference tools**	**Tool categorization nomenclature**	**Practical application**	**Quantitative evaluation?**
Algorithmic Tools for Mining High-Dimensional Cytometry Data	Chester and Maecker, 2015	High-dimensional cytometry data	PCA, viSNE	**4**: ACCENSE, SPADE, FlowSOM, Citrus	Wanderlust	* Dimensionality-reduction techniques	Description of the applications	No
						* Clustering-based analysis		
						* Trajectory detection algorithm		
The end of gating? An introduction to automated analysis of high dimensional cytometry data	Mair et al., 2016	High-dimensional cytometry data	PCA	**5**: SPADE, t-SNE, PSM, Citrus, Phenograph	Wanderlust	* Algorithms for analysis of high-dimensional data	* Describe the applications in other publications * 14-parameter flow cytometry dataset as an practical example and be associated with Mair et al. (2016)	No
Gate to the future: Computational analysis of immunophenotyping data	Mair, 2019	High-dimensional cytometry data	t-SNE	**6**: FlowDensity, FlowType, FlowLearn, FlowSOM, Phenograph, SPADE	None	* Manual gating	Description of the applications	No
						* Algorithm-assisted gating		
						* Algorithm-based clustering		
A Beginner’s Guide to Analyzing and Visualizing Mass Cytometry Data	Kimball et al., 2018	Mass cytometry data	t-SNE, SPADE, others to match tools discussed	**5**: viSNE, SPADE, X-shift, Citrus, PhenoGraph	None	* Automated data analysis	Provide a detailed user-guide using two murine dataset	No
Comparison of Clustering Methods for High-Dimensional Single-Cell Flow and Mass Cytometry Data	Weber and Robinson, 2016	High-dimensional cytometry data	none, only performance comparison	**18**: ACCENSE, ClusterX, DensVM, FLOCK, flowClust, flowMeans, flowMerge, flowPeaks, FlowSOM, FlowSOM_pre, immunoClust, k-means, PhenoGraph, Rcluterpp, SamSPECTRAL, SPADE, SWIFT, X-shift	None	* Clustering methods	Evaluate the tool performance with 6 dataset (4 CyTOF and 2 Flow Cytometry)	Yes (F1 score, running time, expression profiles, stability of the clustering results)
Computational flow cytometry: helping to make sense of high-dimensional immunology data	Saeys et al., 2016	Flow cytometry data	SPADE, FlowMap, FlowSOM, viSNE, PhenoGraph, Scaffold map, DREMI-DREVI	**12**: FLAME, FLOCK, ACCENSE, flowClust, flowMerge, flowMeans, SamSPECTRAL, immunoClust, flowPeaks, FlowSOM meta, HDPGMM, SWIFT, ASPIRE	None	* Methods based on dimensionality reduction techniques	Apply visualization techniques using a manual gated dataset and marker visualization application	No
						* Clustering based techniques		
						* Automated population identification		
						* Biomarker identification		
						* Cell development modeling		
Computational approaches for high-throughput single-cell data analysis	Todorov and Saeys, 2019	Single-cell RNA-seq	PCA, MDS, tSNE, Diffusion maps, SPRING, SPADE, FLOWSOM, Scaffold Maps, FLOWMAP, Phenograph	**7**: SPADE, FLOWSOM, ACCENSE, PhenoGraph, FLOWCAP, ViSNE, Citrus	None	* Visualizing high-dimensional single-cell data	Visualization application using a publicly available scRNA- Seq PBMC dataset	No
						* Dimensionality reduction clustering		
						* Cell type identification		
						* Cell type identification		
						* clustering-based approach		
						* Approaches for modeling gradual transitions		
						* Differential analysis		
						* Cytometry-based approaches		
						* Sequencing-based approaches		
Meeting the Challenges of High-Dimensional Single-Cell Data Analysis in Immunology	Zielinski, 2019	Single-cell RNA-seq	tSNE, PCA, UMAP	**2**: SPADE, FlowSOM	Diffusion pseudotime (DPT); Partition-based graph abstraction (PAGA)	* Linear dimensionality reduction	Visualization and clustering application of a publicly available scRNA-Seq PBMC dataset	No
						* Non-linear dimensionality reduction		
						* Clustering methods; single-cell resolution is lost		
						* Trajectory inference and graph abstraction		
CyTOF workflow: differential discovery in high-throughput high-dimensional cytometry datasets	Nowicka et al., 2017	Mass cytometry data	UMAP, tSNE. MDS	**2**: FlowSOM and ConsensusClusterPlus	None	* Differential analysis * Cell population identification	Detailed data analysis workflow: data pre-processing, clustering, differential analysis and visualization of a publically available CyTOF PBMC dataset	No

*BMC, peripheral blood mononuclear cells.*

Although majority of these papers provide some practical applications of real cytometry data to illustrate how the clustering algorithms function, these review papers have some limitations. (1) Categorization and definition of these computational tools are inconsistent across the review papers. (2) No comprehensive list of clustering tools is provided. With the exception of two reviews that summarized 12 and 18 available tools (Saeys et al., 2016; Weber and Robinson, 2016), majority of the reviews focused on a small subgroup of methods (Chester and Maecker, 2015; Mair et al., 2016; Nowicka et al., 2017; Kimball et al., 2018; Mair, 2019; Todorov and Saeys, 2019; Zielinski, 2019). (3) These reviews do not include comparisons of supervised machine learning algorithms that have gained some popularity in cytometry data analysis. As such, in the current review, we aim to build on the available data to (1) simplify the nomenclature of the categories of available tools, (2) provide a comprehensive comparison of the available unsupervised tools with a real dataset example and systematically review the top six most popular algorithms and (3) review supervised methods that aid in cellular identification.

### Dimensionality Reduction and Visualization Tools Accompanying Clustering Algorithms

Given that cytometry data are multi-dimensional, meaning each individual cell is quantified by multiple parameters (i.e., 30–50 markers for CyTOF experiments), in order to simultaneously visualize these parameters in a low-dimensional manner (i.e., 2 or 3 dimensions), several classical methods have been applied that reduce high dimensional data into low dimensional space (Torgerson, 1952; Wold et al., 1987; Coifman et al., 2005; Maaten and Hinton, 2008; McInnes et al., 2018). Two such common algorithms are principal component analysis (PCA) and t-Distributed Stochastic Neighbor Embedding (t-SNE) (Maaten and Hinton, 2008). PCA linearly transforms the data into orthogonal variables that then can be visualized in low-dimensional space. tSNE employs non-linear transformation of the data to retain probabilities instead of variances and has the benefit of separating individual clusters while preserving the local environment (Maaten and Hinton, 2008). Although tSNE has been widely used to visualize cytometry data, it has a number of limitations including (1) slow computation speed and (2) that the distance between cells cannot be interpreted as cluster relatedness but rather a meaningless variable. Multiple t-SNE based visualization methods have been published to accelerate t-SNE, such as Barnes-Hut t-SNE (Van Der Maaten, 2014) and FIt-SNE (Linderman et al., 2019). Recently another non-linear dimensional reduction technique, uniform manifold approximation and projection (UMAP), has been increasingly used for cluster visualization (McInnes et al., 2018). In a direct comparison between UMAP, t-SNE and other visualization tools, Etienne Becht et al. demonstrated that UMAP performs similar to t-SNE while also preserving the global cluster structure and has superior run time performance (Becht et al., 2019). Although some of the clustering tools described in this review (Table 3), utilize their own unique visualization tools, most of the aforementioned visualization tools can be applied to visualize the clusters generated by any of the algorithms.

**TABLE 3:** Unsupervised clustering tools.

**ID (References)**	**Name**	**Short description**	**Availability**	**Visualization**	**Easy to install and run**	**Cluster # flexibility**	**Reproducible**	**Running time (min)**	**ARI**	**F-measure**

**Unsupervised (compatible with any # of Samples)**										
1. Shekhar et al., 2014	ACCENSE	1. t-SNE dimensionality reduction; 2. k-means or density-based clustering	GUI application	n/a	Yes	No	No	2.48*	0.28*	0.60*
2. Anchang et al., 2014	CCAST	1. identify cell population; 2. refine cluster assignment; 3. estimate a gating scheme by decision tree; 4. optimize the decision tree	R package “CCAST”	Decision tree	Yes	Yes	Yes	77.32	0.71	0.72
3. Chen et al., 2016	ClusterX	1. t-SNE dimensionality reduction; 2. local density estimation; 3. peak detection; 4. clustering assigning	R package “cytofkit”	n/a	Yes	No	Yes	105.14	0.25	0.22
4. Commenges et al., 2018	Cytometree	Implements a binary tree algorithm for clustering	R package “cytometree”	Binary tree	Yes	No	No	12.30	0.08	0.20
5. Ding et al., 2016	densityCUT	1. density estimation; 2. density refinement; 3. local-maxima based clustering; 4. hierarchical stable clustering	R package “densitycut”	n/a	Yes	No	Yes	3.94	0.78	0.34
6. Becher et al., 2014	DensVM	1. t-SNE dimension reduction; 2. density-based peak calling and clustering; 3. SVM classification for less-confident cells	R package “cytofkit”	n/a	Yes	No	No	43.83*	0.71*	0.69*
7. Theorell et al., 2019	DEPECHE	k-means clustering	R package “depecheR”	n/a	Yes	Yes	No	3.46	0.75	0.53
8. MacQueen, 1967; Qian et al., 2010	FLOCK	1. hypergrid creation; 2. identifying dense hyperregions; 3. merging neighboring dense hyperregions; 4. clustering	Available at ImmPort online	n/a	Yes (Need to register at Galaxy)	No (can adjust # of bins and density)	Yes	0.30	0.73	0.65
9. Lo et al., 2009	flowClust	t-mixture models with the Box-Cox transformation	R package “flowClust”	n/a	Yes	Yes	Yes	4.99	0.41	0.43
10. Ye and Ho, 2018	FlowGrid	density-based clustering algorithm DBSCAN with the scalability of grid-based clustering	Github (Python package “FlowGrid”)	n/a	Yes	No (can adjust # of bins and density)	Yes	0.25^	0.54	0.48
11. Aghaeepour et al., 2011	flowMeans	k-means clustering	R package “flowMeans”	n/a	Yes	Yes	Yes	6.01	0.64	0.63
12. Ge and Sealfon, 2012	flowPeaks	1. k-means; 2. Gaussian finite mixture to model the density function; 3. peak search and merging; 4. cluster tightening	R package “flowPeaks”	n/a	Yes	Yes	Yes	0.19	0.64	0.55
13. Van Gassen et al., 2015	FlowSOM	1. self-organization map building; 2. MST building; 3. perform meta-clustering	R package “FlowSOM” and “cytofkit”	MST, Chart plot	Yes	Yes	Yes (if set a seed)	0.19	0.62	0.67
14. Li Y. H. et al., 2017	PAC-MAN	1. partitioning by density-based methods; 2. post-processing	R package “PAC”	n/a	Yes	Yes	Yes	0.35	0.78	0.74
15. Levine et al., 2015	PhenoGraph	1. Construct nearest-neighbor graph; 2. community partitioning	R package “cytofkit”	n/a	Yes	No (Can adjust # of nearest neighbours)	Yes	5.89	0.71	0.78
16. [github]	Rclusterpp	flexible native hierarchical clustering	R package “Rclusterpp”	Hierarchical-structure	Yes (Need to manually download source file)	No	Yes	17.40	0.70	0.71
17. Zare et al., 2010	SamSPECTRAL	Spectral-clustering with data reduction scheme	R package “SamSPECTRAL”	n/a	No (requires manual tuning for optimal results)	Yes	Yes	24.70	0.57	0.33
18. Qiu et al., 2011	SPADE	1. Density-dependent down-sampling; 2. MST construction	R package “spade”	MST	Yes	Yes (given cluster number K, it can create between [k/2,3k/2] clusters	No	2.83	0.58	0.66
19. Mosmann et al., 2014	SWIFT	1. Fit GMM; 2. Refine GMM; 3. agglomerative merging	GUI application by Matlab	n/a	Yes	No (can adjust # of bins and density)	No	20.02*	0.06*	0.29*
20. Samusik et al., 2016	X-shift	1. estimate cell event density; 2. arrange populations by maker-based classification	GUI application	Divisive Marker Trees	Yes	Yes	Yes	35.10	0.65	0.67
21. Sorensen et al., 2015	immunoClust	1. iterative model-based clustering; 2. meta-clustering	R package “immunoClust”	n/a	Yes	No	Yes	82.72	0.29	0.47
22. Flock	k-means	k-means clustering	R base package “stats”	n/a	Yes	Yes	Yes	11.68	0.63	0.63

**Unsupervised (requiring multiple samples)**										
23. Bruggner et al., 2014	Citrus	cluster identification, characterization and regression	R package “Citrus”	n/a	n/a	n/a	n/a	n/a	n/a	n/a
24. Arvaniti and Claassen, 2017	CellCnn	convolutional neural networks	Python 2.7 package on Github	n/a	n/a	n/a	n/a	n/a	n/a	n/a
25. Lun et al., 2017	Cydar	1. cell alignment in hyperspheres in high dimensional space; 2. differential abundance analysis	R package “cydar”	n/a	n/a	n/a	n/a	n/a	n/a	n/a
26. Weber et al., 2018	diffcyt	1. FlowSOM clustering; 2. empirical Bayes moderated tests for differential abundance analysis	R package “diffcyt”	n/a	n/a	n/a	n/a	n/a	n/a	n/a

**Unsupervised (other)**										
27. Pouyan et al., 2016	AUTO-SPADE	1. Fuzzy-C-Mean clustering; 2. Merging clusters using Markov clustering; 3. Integration with SPADE	No tool available							
28. Linderman et al., 2012	CytoSPADE	SPADE clustering	No tool available							
29. Walther et al., 2009	DBM	density based merging (DBM) algorithm	No tool available							
30. Vinh et al., 2009	FLAME	multivariate skew t mixture models	No full tool pipeline available							
31. Finak et al., 2009	flowMerge	1. clustering based on flowClust models; 2. merge clusters	For the downsampled data, number of cluster ranging from 15 to 25 wa applied, but it showed out NA merged result.							
32. Pouyan and Nourani, 2015	Flow-SNE	1. t-SNE data embedding; 2. cluster number estimation; 3. k-means clustering; 4. merging of clusters	No tool available							

*^∗^If the tool cannot complete the running within 3 h, it was applied to a down-sampled data (with 20K cells) for evaluation. ^computing time varies with different setting, but generally fast. MST, minimum spanning tree.*

**FIGURE 2:**
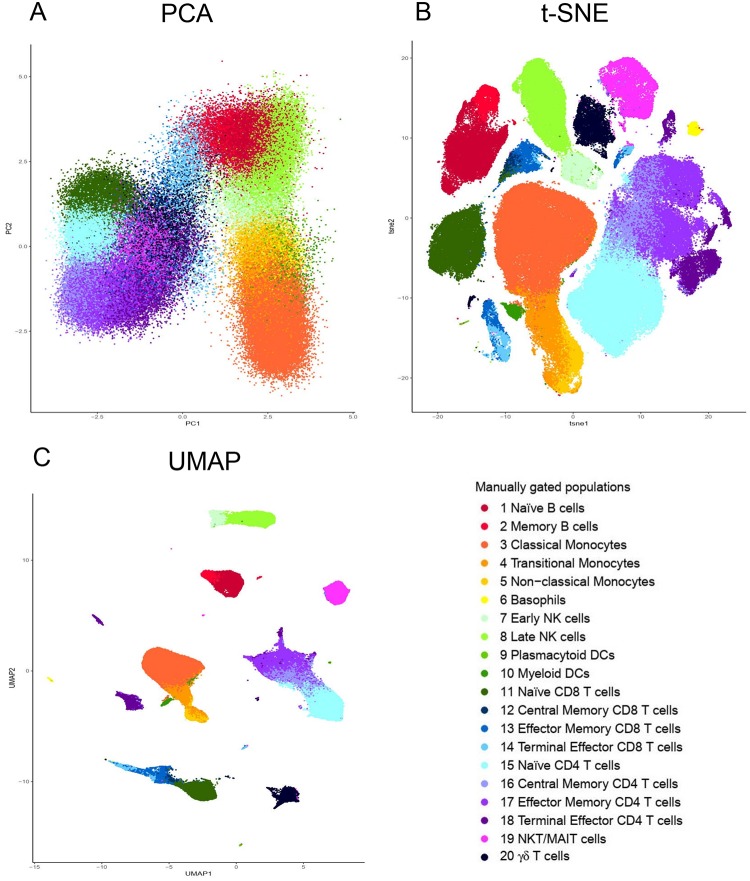
Visualization of dimensionality reduction tools. **(A)** Principal component analysis (PCA); **(B)** t-Distributed Stochastic Neighbor Embedding (t-SNE) and **(C)** Uniform Manifold Approximation and Projection (UMAP). All three dimensional reduction approaches were applied to the same sample from Fluidigm Maxpar Direct Immune Profiling Assay dataset available of Cytobank. The input data was 184,968 CD45 + cells and 21 markers were used (Supplementary Table S2). PCA, t-SNE and UMAP were performed in R using prcomp, Rtsne, and umap functions respectively. Manually gated subpopulations were uniformly colored across all the three plots.

To illustrate this, we have applied PCA, t-SNE and UMAP tools to visualize a peripheral blood mononuclear cell (PBMC) dataset manually gated for twenty immune populations (the generation and details of the dataset are described in a later section) (Figure 2). The twenty identified populations were color coded so that the colors representing a particular population, i.e., dark red for naïve B cells, are conserved across the plots. Both t-SNE and UMAP offer significant separation of the individual clusters beyond that provided by PCA (Figure 2). Moreover, the separation is even more pronounced using UMAP than t-SNE (Figure 2).

In addition to being used as visualization tools, dimensionality reduction methods can also be used to guide manual gating (Eshghi et al., 2019) and have been incorporated into clustering algorithms such as automatic classification of cellular expression by non-linear stochastic embedding (ACCENSE) and density-based clustering aided by support vector machine (DensVM) to reduce the complexity of the dataset as described below.

### Unsupervised Clustering Algorithms

**FIGURE 3:**
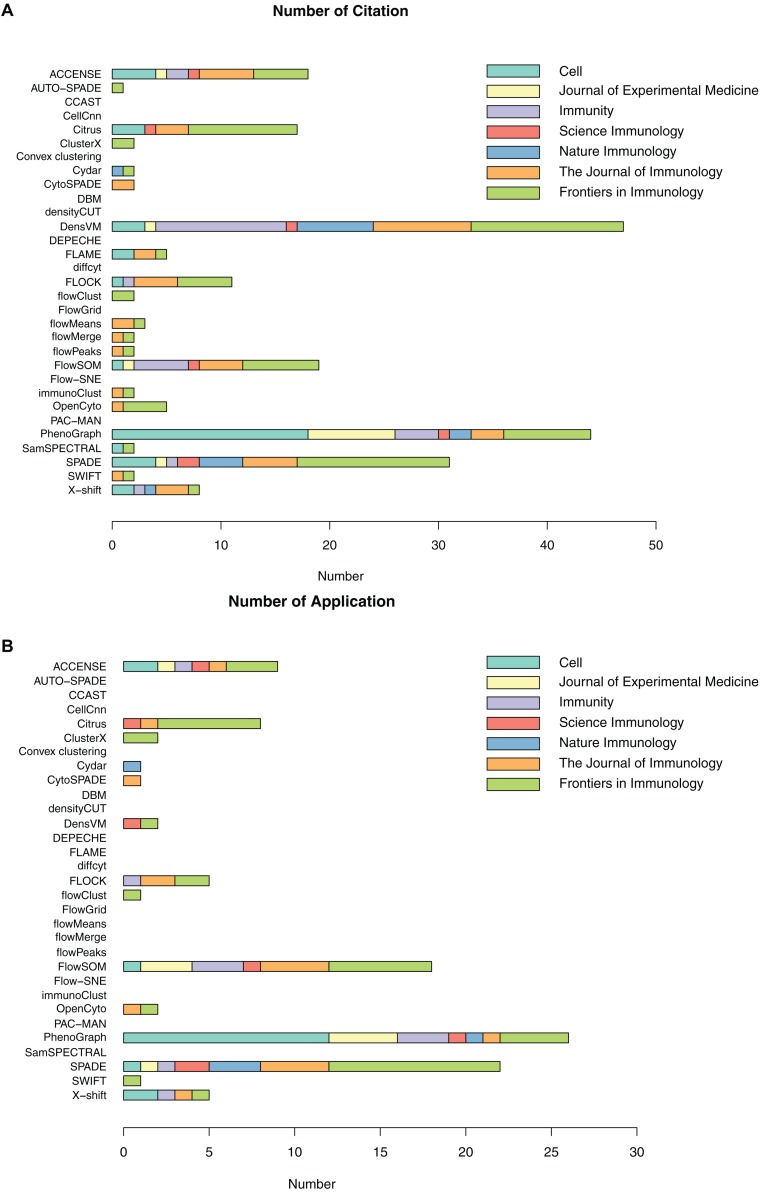
Number of citations and applications for unsupervised clustering tools in seven immunology journals since 2015. **(A)** Number of total citations based on Google Scholar; **(B)** Number of tool applications, only counting citations with real data application by the tools.

As previously introduced, a number of automated, unbiased analysis tools have been developed to assist with clustering of cellular populations in complex datasets. We have grouped these as unsupervised clustering tools. In this review, we summarized 32 of such tools by describing their popularity (Figure 3 and Supplementary Table S1) as well as each tools’ short description, availability, unique visualization platform if offered and easiness to install and run the tool (Table 3). Using Google Scholar, we determined the popularity of each of these methods (Figure 3 and Supplementary Table S1) by summarizing the total number of times each tool has been referenced, or cited, overall and in each of the 7 top immunology journals since 2015 (Figure 3A and Supplementary Table S1). In order to adjust for when the tool was developed, we have also calculated the average annual number of times the tool has been cited (Figure 3A and Supplementary Table S1). Moreover, we have also reviewed the number of times these tools have been directly applied in manuscripts but not just referenced, number of applications across the seven journals and overall since 2015 (Figure 3B and Supplementary Table S1). Based on the tools that have the highest sum of citations and applications overall, we selected the top six tools for a more detailed review outlining their specific advantages and disadvantages.

### ACCENSE (Automatic Classification of Cellular Expression by Nonlinear Stochastic Embedding)

Similar to the other tools discussed in this section, ACCENSE, is a tool for cellular classification of high-dimensional data. It combines dimensionality reduction with density-based clustering to identify sub-populations present in a dataset while retaining the single cell resolution (Shekhar et al., 2014) (Table 3 and Supplementary Table S1).

**FIGURE 4:**
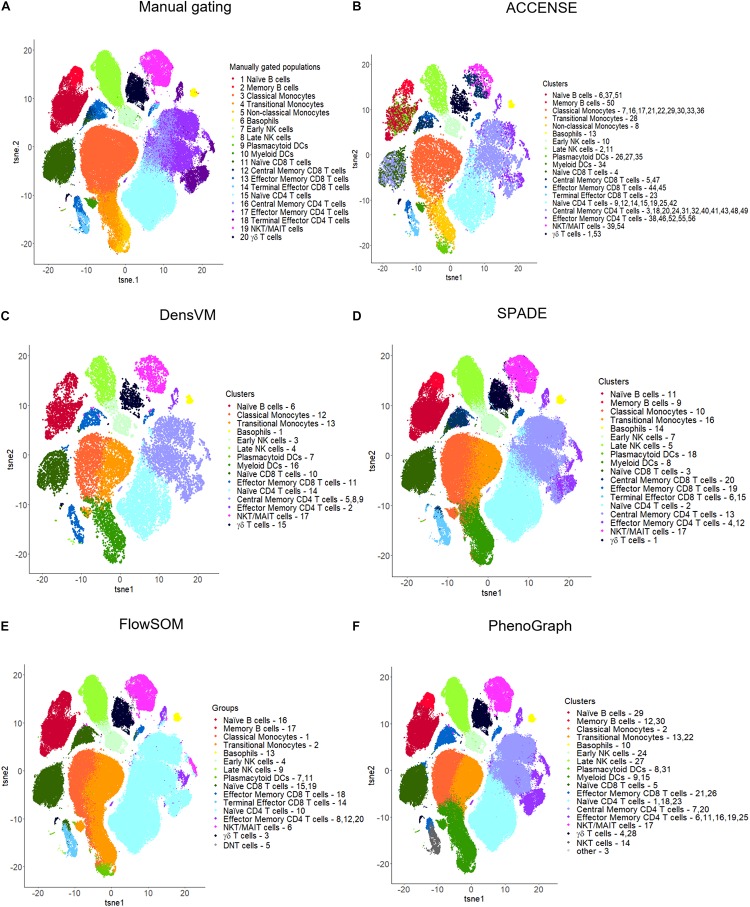
t-SNE visualizations for manual gating and five popular unsupervised clustering tools. **(A)** Manual gating, **(B)** ACCENSE, **(C)** DensVM, **(D)** SPADE, **(E)** FlowSOM and **(F)** PhenoGraph. Manual gating and these five tools were applied to the same data as illustrated in Figure 2. The original t-SNE (Supplementary Figure S4) was altered so that all clustering results were visualized with uniform color coding, where one color represents the same population across all the t-SNE plots. Clusters were manually annotated based on marker expression in each cluster by each clustering method (Supplementary Figure S3). Tools were applied to the full dataset with 180K cells, except for ACCENSE and DensVM, where the data was down-sampled to 20K cells prior to applying the tools as using the full dataset had a running time greater than 3 h. For SPADE and FlowSOM, we set the number of clusters to 20. For ACCENSE, PhenoGraph and DensVM, the number of clusters was automatically optimized by the tool.

In ACCENSE cell subpopulation detection and classification is accomplished in a three-step process. (1) The first step in the process is t-SNE based non-linear dimensionality reduction to reduce the complexity of the data and to improve the speed of the analysis. (2) The second step is to identify cellular subpopulations or clusters. This is accomplished by using kernel-based methodology to determine the local density “peaks” or maxima of the t-SNE generated features and thereby identify the location of the clusters. (3) The final step in the process is to assign the marker expression of each marker in the clusters identified. This is accomplished by performing a phenotypic “coarse-graining” of each individual marker by categorizing its expression pattern as high, intermediate or low in each of the resulting clusters (Table 3 and Figure 4B).

Advantages: The main advantage of ACCENSE is its ease of usability as it is available through the graphical user interface (GUI), an interface that does not require substantial computer skills and makes it attractive for non-computational biologists. Additionally, given that ACCENSE relies on dimensionality reduction with the non-linear t-SNE algorithm, it is able to capture the non-linear phenotypic relationships between cells that are often observed in complex biological systems.

Limitations: However, there are several limitations to ACCENSE use. Although the usability of this tool is improved by having the package available through GUI, as mentioned above, the lack of a script-based package (such as R or Python) that would allow for end-user modifications or streamlining of data analysis is restrictive. Additionally, as is evident in Table 3, where we measured the running time of the same dataset across multiple clustering methods, ACCENSE requires significant down-sampling of the data (from 180,000 to 20,000 cells) to have a reasonable running time. Another limitation of this algorithm is its reliance on cellular density for cluster identification that can miss rare populations. Finally, this algorithm does not contain a user modifiable parameter to control the number of clusters generated. As is evident in Figure 4B, where we show that ACCENSE analysis of a dataset used across multiple clustering algorithms, resulted in over 50 clusters, the default parameter setting tends to detect large number of clusters making biological investigation difficult.

### DensVM (Density-Based Clustering Aided by Support Vector Machine)

The DensVM clustering tool is similar to ACCENSE with some additional modifications to improve the cellular classification (Becher et al., 2014). Similar to the ACCENSE, this algorithm performs dimensionality reduction through t-SNE and detects cellular clusters based on density peaks. However, DensVM contains additional steps to assign cells that are at the periphery of the clusters to the appropriate clusters. This is accomplished in a two-step process, where the algorithm first uses only the cells in any particular cluster whose distances from the peak can be confidently calculated as a training set. The remaining cells that were not assigned to any peak are then grouped into a testing set. The support vector machine (SVM) classifier is then applied to the training set to learn the model and predict the cell cluster assignments for the testing set, where eventually, all cells are grouped into clusters and reported by the algorithm (Table 3 and Figure 4C).

Advantages: Similar to ACCENSE, DensVM takes advantage of the t-SNE algorithm to perform dimensionality reduction allowing for the capture of non-linear phenotypic relationships between cells. One advantage over ACCENSE is that by employing SVM classifiers, cells with confident peak assignment can assist the clustering of uncertain cells. This also reduces the over-clustering seen in ACCENSE (Figure 4B versus 4C).

Limitations: The limitations of DensVM are similar to ACCENSE, except that DensVM can be implemented through an R package allowing users for increased customization and to run larger datasets.

### SPADE (Spanning-Tree Progression Analysis of Density-Normalized Events)

**FIGURE 5:**
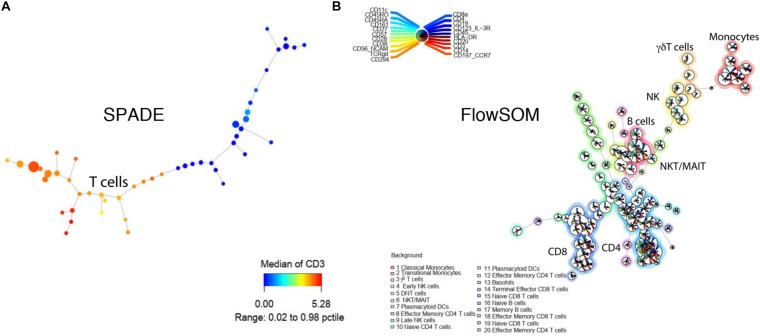
Tree-structure visualization for SPADE and FlowSOM. **(A)** SPADE tree colored by CD3 intensity. **(B)** FlowSOM tree with multiple maker intensities. Both tools were applied to the example from Figure 2.

SPADE is a clustering tool that provides a platform for both cell clustering and data visualization that retains the complex relationship between cellular populations (Murphy and Chused, 1984). The workflow for SPADE analysis consists of four computational steps: (1) First, the algorithm down-samples the data based on cellular density whereby equalizing the representation of rare and major cell populations; (2) It then performs clustering on the down-sampled cells to group cells with similar phenotypes into clusters or “nodes”; (3) It subsequently constructs a minimum spanning tree between all the generated nodes, where each “node” represents a combination of cells with similar properties; (4) As the last step, it maps all the cells in the dataset to the existing clusters, known as up-sampling (Table 3 and Figures 4D, 5A). SPADE offers a unique visualization tool comprised of nodes that are linked to each other by tree like branches where the size of each node corresponds to the number of cells contained within the node (Figure 4A). Moreover, each node can be color coded by the relative expression of a particular marker, i.e., CD3 in Figure 5A, to aid with node identification. This kind of summarized tree-structure plot provides an overview of the cell clusters but will miss the single cell resolution.

Advantages: There are numerous advantages to SPADE. First, SPADE is able to subsample the entire population of cells to achieve an even cell distribution. The algorithm used for the down-sampling of events allows for an equal representation of both dominant and rare populations. Additionally, the spanning tree structure of the output permits the identification of the hierarchical relationship between the various clusters identified. For example, cellular populations that are similar to one another are found in the same branch of the dendrogram, while the subtypes located at different branches are minimally related. The algorithm is user friendly and highly modifiable so that the number of nodes and the similarity of cells within the node can easily be adjusted.

Limitations: However, there are a number of limitations to this method. Because the program color codes the entire dendrogram to represent the relative expression of each marker in every node, visualization of more than one marker at a time is challenging. Additionally, although theoretically SPADE should be able to detect rare populations, as reported by Weber and Robinson (2016), SPADE did not perform well to balance precision and recall for rare population detection. However, some of the issues have been overcome by the updated version of SPADE (Qiu, 2017).

### FlowSOM (Self-Organizing Map Clustering)

FlowSOM uses consensus clustering to organize cells and aims to analyze cytometry data with Self-Organizing Maps (SOM) (Van Gassen et al., 2015). FlowSOM clustering algorithm includes four computational steps: (1) Scaling within each marker; (2) Building up a SOM with nodes representing the overall composition of neighboring cells and assigning cells to the closest node; (3) Visualizing the SOM by building a minimal spanning tree to connect the nodes into a graph; (4) Calculating the meta-clustering of the nodes and automatically suggesting the best number of clusters for each particular dataset (Table 3 and Figure 4E).

Advantages: One of the main advantages of FlowSOM is that it is one of the fastest clustering tools available. In our direct comparison, it had the shortest running time of 0.19 min for 1,800,000 events, only comparable to flowPEAK (Table 3). Additionally, FlowSOM nodes can be visualized by star charts that simultaneously show the mean value of multiple markers in a pie chart (Figure 5B), greatly facilitating with cluster or node identification.

Limitations: FlowSOM improves marker visualization with the ability to visualize multiple simultaneously allowing for easier population identification. However, if too many markers are visualized at the same time, the interpretation can still be difficult (Figure 5B).

### PhenoGraph (an Algorithm for Defining Cellular Phenotypes in High-Dimensional Single-Cell Data)

The PhenoGraph algorithm is another automated clustering tool that identifies clusters of phenotypically similar cells (Levine et al., 2015). Cellular clusters are generated in a two-step process. (1) First, the tool defines the nearest neighbors for each cell by using Euclidean distance, and then constructs a graph where each node represents an individual cell and each edge represents the number of shared neighbors. (2) PhenoGraph then partitions the graph into distinct populations based on the Louvain community detection method where cells with similar phenotypes are clustered together. In this way, each community represent a unique population of cells with similar phenotypic features, and the connections between communities represent the correlation between the various populations (Table 3 and Figure 4F).

Advantages: PhenoGraph is able to retain the single cell nature of the data and uses the relationship between cells to identify communities. Additionally, PhenoGraph is especially powerful for datasets containing a large number of samples or large number of cells per samples, where it can efficiently perform clustering of hundreds of thousands or even millions of cells without the need for down sampling. Finally, another advantage of this tools is its ability to determine cluster number automatically, without any known prior information and without producing too many clusters (Table 3 and Figure 4F), i.e., 31 clusters produced for the example dataset.

Limitations: The PhenoGraph clustering results can be visualized by t-SNE, PCA, or a heatmap, however, the community graph data presented in the original paper (Ferrer-Font et al., 2019) is not available in the current version of R package. In the current R package, the number of clusters are automatically determined by the algorithm. Although PhenoGraph is designed to optimize cluster number automatically, because this parameter cannot be altered by the end user, it sometimes results in a large number of clusters than might not have biological significance (Figure 4F). The algorithm performs efficiently when users apply default settings, but the time needed for cluster generation increases as users alter these settings (Table 3 and Figure 4).

### Citrus (Cluster Identification, Characterization, and Regression)

Citrus is a data-driven approach to identify cell populations present in high-dimensional data sets and correlate them to particular outcomes (Bruggner et al., 2014). It accomplishes this by performing first unsupervised cell clustering followed by supervised prediction modeling to identify cell responses that are highly correlated with experimental endpoint in a three-step process. (1) Citrus first conglomerates individual cells from all samples together into one dataset and then performs hierarchical clustering of all the cells. The algorithm then filters out all clusters below a minimal threshold (this can be adjusted) and uses the remaining clusters for subsequent analysis. (2) As the second step, individual cells are then reassigned back to the original samples and Citrus generates a matrix of descriptive features and metadata for each sample. (3) Finally, Citrus builds a regression model of the data based on the feature matrix and the metadata provided to correlate cellular clusters with the experimental endpoint (Table 3).

Advantages: Compared with other clustering method, Citrus can not only identify cell subpopulations, but can also detect which cellular subsets correlate with the experimental endpoints of interest. This tool is provided both as an R package and easy-to-use GUI, making it convenient for most users.

Limitations: One of the main limitations of Citrus is its inability identify less frequent cell populations as one of the steps in the algorithm filters out cell clusters below a minimum cluster threshold. Additionally, Citrus requires at least 8 samples in each experimental group for optimal performance and this is not always possible to obtain.

### Overall Summary of Unsupervised Clustering Methods

As shown in Table 1, unsupervised clustering tools as a group have the following advantages: (1) Compared with manual gating, these clustering pipelines are unbiased and fully automated; (2) Although the computing time is variable and depends on the particular tool used, overall the computing speed is generally fast. (3) Unlike manual gating that requires prior-knowledge by the end user, these clustering tools perform “blindly” that allows for the detection of novel cell types and rare cell populations. This is accomplished in some methods by specific density-dependent dimensionality reduction, where down-sampling allows for equal representation of rare and abundant cell types. (4) Some algorithms can suggest the optimal number of cell clusters to be generated such as PhenoGraph, DensVM, and ClusterX, while other algorithms such as SPADE, FlowSOM, and CCAST are also able to accept manual input for the desired number of clusters to be generated (Table 3).

However, there are a number of limitations to the current unsupervised clustering tools that potentially can be improved. First, although these algorithms cluster cells into subpopulations, the identification of these clusters requires annotation by the end-user, that can be time consuming, biased and prone to errors. To address this issue, tools such as the combination of MEM (Diggins et al., 2017) + flowCL (Courtot et al., 2015) can automatically identify the marker signatures and match with cell ontogenies of known cell types. Furthermore, this has led to the development of supervised clustering tools that can not only cluster the cells but also annotate the resulting clusters. These will be discussed below. Another disadvantage of the currently available tools is the inconsistency in their implementation pipeline, where some methods are primarily based on a GUI application while others only have script-based packages available with very few allowing for both possibilities. GUI-based packages make the analysis more user-friendly. However, tools only using GUI are usually less efficient for processing large datasets and offer less flexibility in adapting the preset parameters to the end user’s needs. On the other hand, tools that only have script-based packages available are less accessible to users with limited computational training. As such, it would be beneficial for clustering tools to offer both pipelines.

### Supervised or Semi-Supervised Clustering Tools

Recently supervised and semi-supervised clustering algorithms have been developed that allow simultaneous cellular clustering and cluster annotations (Table 4). Based on the additional information required for the implementation of these methods, they can be classified into the following subtypes: (1) Supervised machine learning clustering algorithms that rely on annotated training sets as input, to “train” them for patterns associated with each cluster to predict cluster identity of new samples. Some of these tools use computer learning algorithms such as linear discriminant analysis (LDA) (Abdelaal et al., 2018) or neural networks (Li H. et al., 2017) to apply the patterns extracted from the training sets to annotate cells from a new dataset. (2) The semi-supervised clustering algorithms incorporate user provided marker matrix of known marker associations with particular cell types (Lee et al., 2017; Ji et al., 2018) to guide cellular clustering and identification. These marker matrices are composed of marker expression patterns in various cell types that serve as a cluster dictionaries indicating whether the markers are negative, positive or ignorable for each cell types. Others, for example openCyto (Finak et al., 2014), rely on a gating template hierarchy to facilitate with cluster annotation. Yet another tool, flowLearn (Lux et al., 2018) aligns markers’ density from manually gated data to other samples to estimate the gating threshold. These methods provide alternatives to unsupervised clustering tools with an example of each type is outlines below.

**TABLE 4:** Supervised clustering tools.

**ID (References)**	**Name**	**Short description**	**Availability**	**Additional information**	**Implementation**	**Running time (min)**	**ARI**	**F measure**	**Notes**
1. Finak et al., 2014	OpenCyto	A method mimicking manual gating by incorporating information from a gating template	R package “openCyto” available on Bioconductor	Gating template, can be a complete table or added inline one cell type at a time	Tututorials avaialble, preparing the gating template is challanginng	Fast (running time depends on the choice of algorithms in the gating template)	Not evaluated	Not evaluated	We did not evaluate ARI and F measure because OpenCyto is not fully automated, it needs user”s supervision and fine parameterization.
2. Li H. et al., 2017	DeepCyTOF	Uses training data to predict cell types based on deep learning techniques	Python, Github	Training data	Time consuming to understand examples scripts and adapt it to your own data	1.36	0.96	0.93	50% of the cells in the sample were randomly chosen as training sample
3. Abdelaal et al., 2018	CyTOF Linear Classifier	Uses training data to predict the cell types based on linear discriminant analysis (LDA)	R, Matlab, Github	Training data	Easy to run	0.12	0.91	0.92	50% of the cells in the sample were randomly chosen as training sample
4. Lee et al., 2017	ACDC	Uses a marker matrix information to predict the cell types based on semi-supervised learning techniques	Python package (Bitbucket)	Markers matrix	Time consuming to understand examples scripts and adapt it to your own data	24	0.81	0.77	–
5. Ji et al., 2018	MP (Mondrian)	Uses a marker matrix to predict cell types through a Bayesian model	Python, github	Markers matrix	Time consuming to understand examples scripts and adapt it to your own data	109	0.55	0.49	50% of the cells in the sample were randomly chosen as training sample
6. Lux et al., 2018	flowLearn	Uses gates from training data to predict gating threshold in other samples through a density alignment	R package flowLearn, github	Training data	Not fully automate, gate one marker at a step.	Fast for predicting one threshold at each step	Not evaluated	Not evaluated	We did not evaluate ARI and F measure because flowLearn is not fully automated and needs user’s supervision.
									

### DeepCyTOF

DeepCyTOF is an example of a supervised machine learning algorithm that integrates deep machine learning into automatic cell population gating (Li H. et al., 2017). This algorithm relies on manually gated examples for cellular population identification in new samples. The algorithm accomplishes cluster identification in a three-step process. (1) It first uses the provided manually gated and annotated data as a training set. (2) It then performs denoising and data calibration of the new data with the training sets to reduce batch effects and (3) performs cellular classification of the new data based on the training set provided through a feed-forward neural network model (Zell, 1994).

Advantages: One advantage of DeepCyTOF in addition to that offered by the supervised machine learning algorithms at large, is that it is able to calibrate new data to training sets, limiting the batch to batch variation that can happen in data that are not simultaneously generated.

Limitations: One of the limitation specific to DeepCyTOF is that it relies on an annotated training set of data. Although this step allows for cluster annotation, it also introduces end-user bias in requiring manually gated cellular populations. In addition, similar to all other supervised learning approaches, DeepCyTOF is not able to identify novel cell populations as they are not predefined in the training set.

### ACDC (Automated Cell Type Discovery and Classification)

ACDC is an example of a semi-supervised clustering algorithm that incorporates a user-specified marker matrix to identify cellular cluster (Lee et al., 2017). It then uses this matrix to define cellular populations based upon particular markers. The marker matrix is composed of all markers used in a dataset with assigned values for each marker in various defined cellular populations where marker assignment can be -1, 1, or 0 (never present, present or unrelated). ACDC then converts the marker matrix into landmark points which represent cellular population’s fingerprints, and the semi-supervised learning algorithm is implemented through a random walk process where each individual cell is classified to belong to one of the predefined populations or is labeled as “unknown.”

Advantages: One of the advantages specific to ACDC, is that it classifies ambiguous cells into an “unknown” group, which although cannot be directly identified, can be exported for further investigation.

Limitations: One limitation of ACDC is that the markers that are used to define cellular populations are binary. As a large number of markers do not have a binary expression (expressed or not expressed) but rather are expressed on a continuum, this method therefore either excludes these markers from defining the cellular populations or requires the end user to arbitrarily assign expression cut-offs for those markers.

## Overall Summary of the Supervised and Semi-Supervised Automated Gating Approaches

The supervised and semi-supervised machine learning clustering algorithms combine merits offered by manual gating and automated clustering algorithms and provide sophisticated methods for the development of reproducible and automated gating and cluster identification pipelines. Compared with the unsupervised clustering methods, they not only automatically group cell into clusters, but also provide annotation for those clusters. The end-user prespecified gating strategies either in the form of marker tables or in pre-gated data sets ensures that the algorithms’ uses user accepted methods to define cellular populations. However, this process also introduces subjectivity and bias absent from unsupervised gating tools. Additionally, the process relies on the end-users’ prior knowledge and is labor intensive. Another limitation of most of these methods is that they lack user friendly interface and rely on users’ ability to program based on provided examples, limiting their use for those who do not have extensive computational skills. Finally, identification of rare and novel cell types is still challenging for these methods.

### A Practical Application

To evaluate and compare the performance of the various unsupervised and supervised clustering tools, we applied these algorithms to a public dataset [Fluidigm_Maxpar Direct Immune Profiling Assay_201325_Gating Example_v1.0 (Public)] downloaded from Cytobank (Kotecha et al., 2010) for a total of 32 unsupervised and 6 supervised/semi-supervised clustering tools. This dataset included CyTOF data on 42 human peripheral blood mononuclear cells (PBMCs) samples, where we randomly chose one PBMC sample (HulmmProfiling_S1_PBMC_1) and applied it to all the various methods. After processing the data to filter out beads, dead cells and doublets, there were 184,968 cells that remained in the dataset. We then manually gated the dataset using 21 markers to predefine 20 unique cell subpopulations (Supplementary Figure S1 and Supplementary Table S2) and used these manually gated populations as our reference or “truth” for comparison of the clustering algorithms performance. These tools were compared across four categories (Tables 3 and 4): (1) tool running time; (2) if the number of clusters can be altered or is predefined; (3) reproducibility of the results when repeat five times and finally (4) we also measured the consistency between the clustering algorithms and the manual gating by two separate measures: adjusted rand index (ARI) (Rand, 1971; Hubert and Arabie, 1985; Vinh et al., 2009) and F-measure (Hubert and Arabie, 1985; Sasaki, 2007; Tables 3 and 4). ARI is a measurement for the similarity between two clusters, where ARI = 1 represents two clusters that are the same, where an ARI value close to 0 (or even negative value) means high dissimilarity between the two clusters (Rand, 1971; Vinh et al., 2009). ARI is calculated by the adjustedRandIndex function of mclust package in R. The F-measure (or F1 score) is a tool to measure similarity between prediction and truth, which can balance precision and recall. Both ARI and F-measure are calculated using R (3.6.1).

Precision=true positives/(true positives + false positives)

Recall=true positives/(true positives + false negatives)

F-measure=2×Precision×Recall/(Precision + Recall)

For a given cluster in the manual gating (serving as truth), we calculate the F-measure between this true cluster and all the clusters reported by the methods. The highest F-measure was regarded as the best match between this true cluster and predicted clusters and was used as the F-measure value for this given true cluster. For each true cluster, we repeated this step to get the F-measure for all the true clusters. We then averaged the F-measure values across all the true clusters and used this value to report in Tables 3 and 4 (Weber and Robinson, 2016). Of note, we did not intend for the comparison ARI and F-measure results to be conclusive as we only used one sample for the data generation. They are merely used here for direct comparison between tools. More comprehensive evaluation is beyond the scope of this paper.

Table 3 provides the detailed results for the comparison of the unsupervised clustering methods. We were able to apply the model dataset to 21 out of the 32 methods listed in Table 3 as some of the methods are no longer available or could not be implement, such as CytoSPADE (Linderman et al., 2012) and FLAME (Pyne et al., 2009). Among those packages that we could successfully implement, most of the tools had available R packages and were easy to implement. For cluster generation, we used all default parameters, unless the number of clusters could be specified in which case we had set it to 20 or the number of cell populations identified by manual gating. The running time ranged from 0.19 min to over 3 h (Table 3). Out of all the supervised algorithms tested FlowSOM, PAC-MAN and FlowPEAKS had the fastest running time. On the other hand, DensVM, ACCENSE and SWIFT were very slow (over 3 h) and required down sampling to 20,000 cells to accelerate the clustering. The results from DensVM, SPADE, and DEPECHE were not consistent across different runs, while FlowSOM generated reproducible results by setting a fixed seed. Whereas methods such as PhenoGraph, CCAST, and Rclusterpp were consistent across multiple runs. PhenoGraph, PAC-MAN, FLOCK, DensVM, CCAST, and Rclusterpp resulted in clusters that were the closest to manual gated clusters with high ARIs and F measures (Table 3). PhenoGraph and PAC-MAN had the highest ARIs and F measures among all the tools tested (Table 3).

The results for the comparison of supervised tools are shown in Table 4. For those methods requiring a training dataset such as DeepCyTOF, CyTOF linear classifer and flowLearn, we set aside half of the cells in the dataset as the “training” set and used the remaining cells in the dataset as the “validation” dataset. As such the performance measure of these tools might not be reflective of what would be obtained with an unrelated dataset. Many of the supervised methods did not have easy to use packages available, and relied on users to write their own code based on provided examples. DeepCyTOF (1.36 min) and CyTOF linear classifier (0.12 min) required the shortest amount of time to run (Table 4). As expected, overall the semi-supervised and supervised tools had higher ARI and F1 measures compared to unsupervised clustering methods, since they incorporate user defined gating strategies into the clustering and gating process.

For consistency of the results, we chose t-SNE plots for visualization of the clustering results of all the tools tested (Figure 4 and Supplementary Figures S2, S4). All visualizations were generated using R (3.6.1). Figure 4 and Supplementary Figure S4 shows the cell clusters resulting from manual gating and the five most popular unsupervised clustering tools (ACCENSE, DensVM, SPADE, FlowSOM, and PhenoGraph) described in more detail in the previous sections. We did not visualize Citrus generated data, as we only used one sample and Citrus requires a large input. Cellular population of a similar phenotype have been color coded across all six of the t-SNE plots for ease of comparison based on the associated heatmaps displaying mean marker expression level across all clusters (Supplementary Figure S3). As described in the previous sections, since the cluster number cannot be adjusted in ACCENSE (56 clusters) and PhenoGraph (31 clusters), they resulted in the highest number of clusters, whereas SPADE and FlowSOM whose cluster number can be defined produced 20 clusters each (Figure 4 and Supplementary Figure S4). DensVM resulted in 17 clusters (Figure 4 and Supplementary Figure S4). Of the five top tools used, all of the unsupervised algorithms contained at least one cluster that corresponded to one of the twenty reference populations identified by manual gating. However, in a number of clusters, the marker expression patterns of reference cell types and those in the obtained clusters did not match perfectly. In ACCENSE, we observed a significant over splitting of the cells, resulting in many small clusters (Figure 4 and Supplementary Figure S4).

### Challenges and Future Directions

As reviewed in this manuscript, many methods, including unsupervised and supervised clustering tools have been developed in recent years to aid the analysis of high-dimensional cytometry data. Many of these methods have been adopted by the community and have significantly improved our understanding of immune cell populations (Chester and Maecker, 2015; Mair et al., 2016; Saeys et al., 2016; Weber and Robinson, 2016; Kimball et al., 2018; Mair, 2019; Todorov and Saeys, 2019; Zielinski, 2019). These automated gating algorithms can be implemented on large data sets, and have the potential to detect novel cell types and cellular relationships not easily identifiable by manual gating. The most popular methods are ACCENSE, DensVM, SPADE, FlowSOM, PhenoGraph, and Citrus.

In this manuscript, we implemented a significant proportion of these methods using a test PBMC CyTOF dataset and directly compared their performance to manual gating. Although we have opted to use a CyTOF dataset for the comparison, any cytometry data can be similarly used. Runtime varied drastically between the various methods tested, but FlowSOM, FlowPEAK, and PAC-MAN had the shortest computing time of the unsupervised algorithms and DeepCyTOF and CyTOF linear classifiers performed the fastest of the supervised tools. Several of the unsupervised clustering algorithms, such as PhenoGraph and PAC-MAN, had reproducible results and compared well to manually gated results. As expected, supervised methods in general had higher ARIs and F measure than the unsupervised methods with DeepCyTOF achieving the highest ARI and F measure among all the supervised methods. However, more comprehensive results are needed to validate our findings.

In respect to usability of the available tools, many of the unsupervised clustering algorithms had easy to use packages available, although some of them were either no longer available or we were not able to run successfully. On the other hand, the supervised gating methods were more difficult to implement, as they required additional information such as a marker matrix or samples with gated populations for input and/or did not have easy to use packages available. For example, methods such as DeepCyTOF and CyTOF linear classifier require non-trivial programming skills to implement.

Although these methods have significantly improved our ability to work with multi-dimensional data, based on our reviews and quantitative analysis, there remain several challenges that should be addressed with future tool development. Many of the clustering tools rely on the end-user to have significant computational skills, limiting their availability for a wider audience and as such future tools would benefit from incorporating a GUI or shiny app interphase along with R/python scripts for wider appeal. Current methods are not fully automated and still rely on significant user input. Unsupervised clustering methods rely on manual labeling of clusters to identify the populations, whereas supervised auto-gating methods need prior information such as a user specified marker matrix or a manually gated training dataset. Additionally, rare and novel population identification is challenging especially for the supervised clustering tools. It would be beneficial for future tools to address these challenges by incorporating built in cluster identification methods and those that can infer potential novel populations based on previously known data. In order to reduce computing time for some computationally demanding methods, subsampling of cells has been a popular approach. Analysis of impact of subsampling has not been fully studied, especially on clustering accuracy and ability to identify rare population.

As these tools gain popularity and become routinely applied to large datasets such as patient monitoring in clinical trials other unique challenges arise that should be addressed by the next generation of tools. These tools should be able to handle large datasets containing millions of cells per file and large number of files. Similarly, studies comprised of data collected over multiple cytometry runs are prone to batch effect that needs to be incorporated into the new algorithms. Batch effect, or technical variability between experiments, if not accounted for can result in overestimation of the heterogeneity of the sample and identification of “false” clusters of cells, where two or more clusters are actually of the same phenotype but are represented by unique clusters secondary to non-biological phenomena. Additionally, there are unique challenges inherent to high-dimensional flow cytometry data that need to addressed by future tools (Mazza et al., 2018) by incorporating algorithms that can compensate for a number of parameters that can introduce variability to flow data such as background fluorescence and spreading error (Roederer, 2001), inability to resolve a true positive population due to “spreading” of the negative populations.

Finally, these tools have been routinely applied to cluster immune cells, where markers that define particular cellular identities are well defined, e.g., CD3 for T cells, CD19 or CD20 for B cells. However, using these tools to cluster non-immune cells or a combination of immune and non-immune cells possess its own challenges as markers that define unique cellular populations are not as well defined. Although in principle, all the clustering algorithms should function similarly irrespective of the particular markers present, classifying the identity of the generated non-immune cellular clusters is much more challenging.

Despite current challenges, an increasing number of user-friendly clustering methods have been developed. Future tool development should focus on developing methods with modifiable user-friendly interfaces, better accuracy and reproducibility, higher computational efficiency and decreased human intervention. Multidisciplinary collaboration is needed to address these challenges and to push the automated clustering tools into the next generation that is able to utilize the high-throughput cytometry technologies, minimize user burdens and give more insights into population identification.

## Author Contributions

PL and SL wrote the manuscript and performed data analysis. LK and GT supervised the work and edited the manuscript. YF, XX, and JZ performed the benchmarking analysis. All the authors approved the final version of the manuscript.

## Conflict of Interest

The authors declare that the research was conducted in the absence of any commercial or financial relationships that could be construed as a potential conflict of interest.

## Supplementary Material

FIGURE S1 Manual gating hierarchy for the major populations (20) used in the real data application section.

FIGURE S2 t-SNE visualizations for the clustering results by **(A)** CCAST, **(B)** ClusterX, **(C)** densityCUT, **(D)** DEPECHE, **(E)** FLOCK, **(F)** flowClust, **(G)** FlowGrid, **(H)** flowMeans, **(I)** flowPeaks, **(J)** PAC-MAN, **(K)** Rclusterpp, **(L)** SamSPECTRAL, **(M)** SWIFT, **(N)** X-shift, **(O)** immunoClust and **(P)** k-means. These tools were applied to the same dataset as in Figures 2, 4, 5. Colors are not matched across the tools in this figure. Tools were applied to the full data with 180K cells, except for SWIFT where it was down sampled to 20K cells. Clustering result for Cytometree is not plotted because it generated more than 1,000 clusters.

FIGURE S3 Marker expression heatmap for the clustering results Figure 4 and Supplementary Figure S4. **(A)** Manual gating, **(B)** ACCENSE, **(C)** DensVM, **(D)** SPADE, **(E)** FlowSOM and **(F)** PhenoGraph. Manual gating and the five popular tools were applied to a real data to perform cell subpopulation identification. Their results are visualized by heatmap with rows representing markers and columns for clusters. Color darkness level (refer to color bar) indicates the marker median metal intensity across all the cells within a given cluster.

FIGURE S4 t-SNE visualizations for manual gating and five popular unsupervised clustering tools. **(A)** Manual gating, **(B)** ACCENSE, **(C)** DensVM, **(D)** SPADE, **(E)** FlowSOM and **(F)** PhenoGraph. Clusters for each tool are painted with unique colors. Manual gating and the five popular tools were applied to the same data as Figure 2. Tools were applied to the full dataset with 180K cells, except for ACCENSE and DensVM, where the data was down-sampled to 20K cells prior to applying the tools as using the full dataset had a running time greater than 3 h. For SPADE and FlowSOM, we set the number of clusters to 20. For ACCENSE, PhenoGraph and DensVM, the number of clusters was automatically optimized by the tool.

TABLE S1 **(A)** Unsupervised clustering tools citations. **(B)** Unsupervised clustering tools applications.

TABLE S2 Manual gating markers.
