# Supplementary material for: Recent Advances in Computer-Assisted Algorithms for Cell Subtype Identification of Cytometry Data
Source: Front Cell Dev Biol. 2020 Apr 28;8:234. doi: 10.3389/fcell.2020.00234 (PMC7198724; doi:10.3389/fcell.2020.00234)
Supplement: FIGURE S1 — Manual gating hierarchy for the major populations (20) used in the real data application section. [file Image_1.pdf]

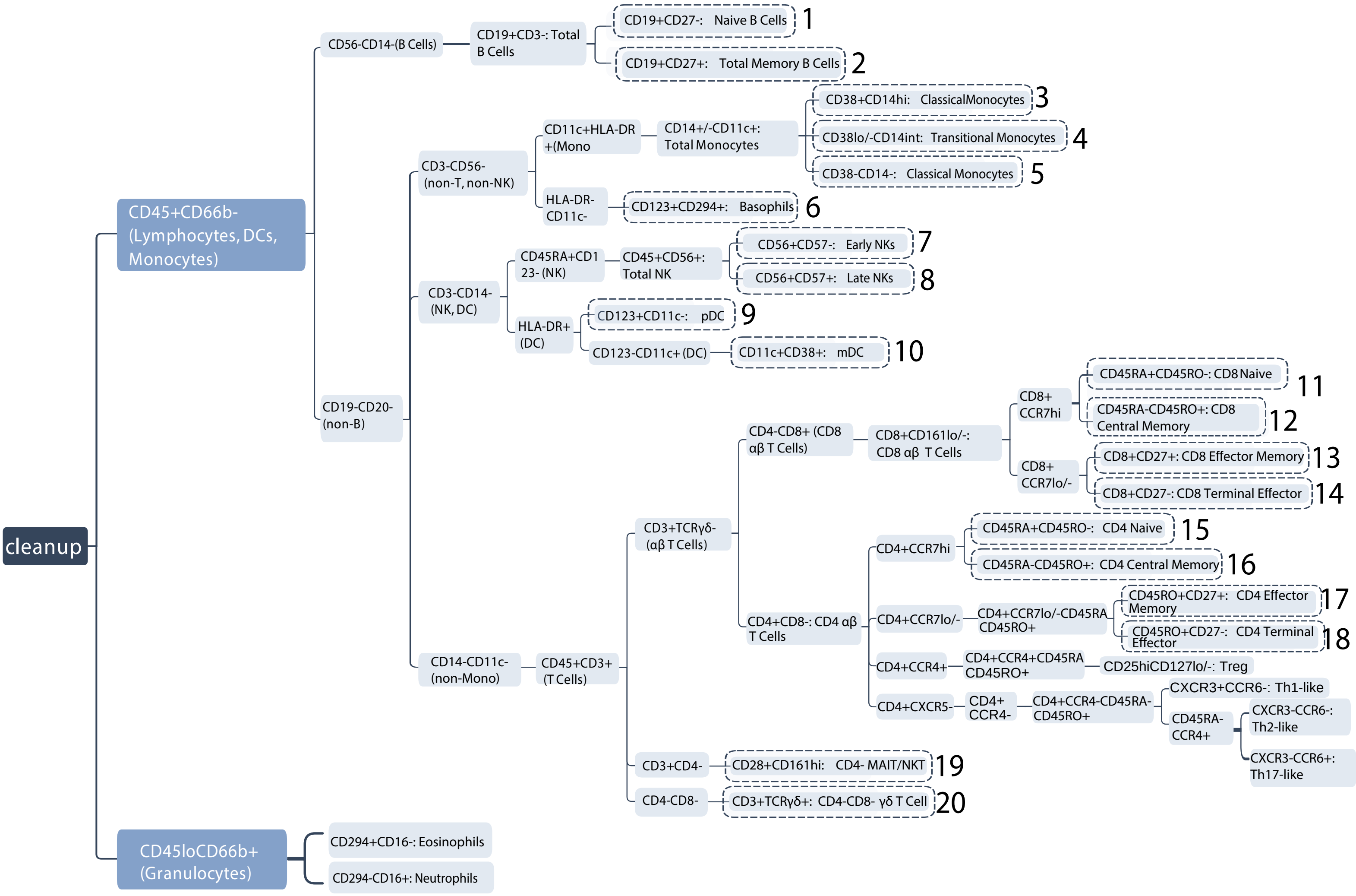
cleanup
CD45+CD66b- (Lymphocytes, DCs, Monocytes)
CD56-CD14-(B Cells)
CD19+CD3-: Total B Cells
CD19+CD27-: Naive B Cells
1
CD19+CD27+: Total Memory B Cells
2
CD3-CD56- (non-T, non-NK)
CD11c+HLA-DR +(Mono
CD14+/-CD11c+: Total Monocytes
CD38+CD14hi: ClassicalMonocytes
3
CD38lo/-CD14int: Transitional Monocytes
4
CD38-CD14-: Classical Monocytes
5
HLA-DR- CD11c-
CD123+CD294+: Basophils
6
CD3-CD14- (NK, DC)
CD45RA+CD123- (NK)
CD45+CD56+: Total NK
CD56+CD57-: Early NKs
7
CD56+CD57+: Late NKs
8
HLA-DR+ (DC)
CD123+CD11c-: pDC
9
CD123-CD11c+ (DC)
CD11c+CD38+: mDC
10
CD19-CD20- (non-B)
CD14-CD11c- (non-Mono)
CD45+CD3+ (T Cells)
CD3+TCRγδ- (αβ T Cells)
CD4-CD8+ (CD8 αβ T Cells)
CD8+CD161lo/-: CD8 αβ T Cells
CD8+ CCR7hi
CD45RA+CD45RO-: CD8 Naive
11
CD45RA-CD45RO+: CD8 Central Memory
12
CD8+ CCR7lo/-
CD8+CD27+: CD8 Effector Memory
13
CD8+CD27-: CD8 Terminal Effector
14
CD4+CD8-: CD4 αβ T Cells
CD4+CCR7hi
CD45RA+CD45RO-: CD4 Naive
15
CD45RA-CD45RO+: CD4 Central Memory
16
CD4+CCR7lo/-
CD4+CCR7lo/-CD45RA CD45RO+
CD45RO+CD27+: CD4 Effector Memory
17
CD45RO+CD27-: CD4 Terminal Effector
18
CD4+CCR4+
CD4+CCR4+CD45RA CD45RO+
CD25hiCD127lo/-: Treg
CD4+CXCR5-
CD4+ CCR4-
CD4+CCR4-CD45RA- CD45RO+
CXCR3+CCR6-: Th1-like
CD45RA- CCR4+
CXCR3-CCR6-: Th2-like
CXCR3-CCR6+: Th17-like
CD3+CD4-
CD28+CD161hi: CD4- MAIT/NKT
19
CD4-CD8-
CD3+TCRγδ+: CD4-CD8- γδ T Cell
20
CD45loCD66b+ (Granulocytes)
CD294+CD16-: Eosinophils
CD294-CD16+: Neutrophils
