# Supplementary figures and images for: Recent Advances in Computer-Assisted Algorithms for Cell Subtype Identification of Cytometry Data
Source: Front Cell Dev Biol. 2020 Apr 28;8:234. doi: 10.3389/fcell.2020.00234 (PMC7198724; doi:10.3389/fcell.2020.00234)

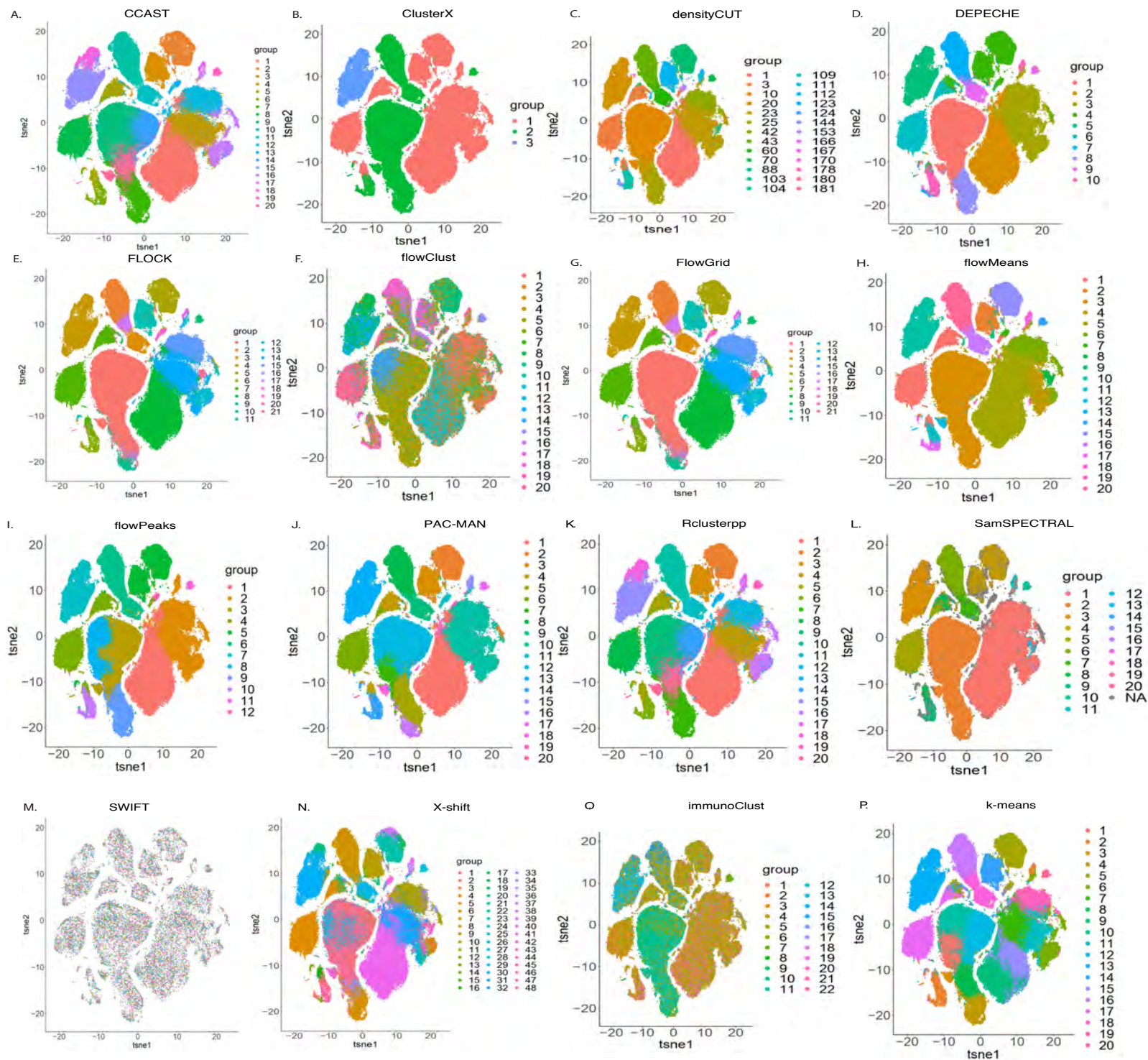

Supplement: FIGURE S2 — t-SNE visualizations for the clustering results by (A) CCAST, (B) ClusterX, (C) densityCUT, (D) DEPECHE, (E) FLOCK, (F) flowClust, (G) FlowGrid, (H) flowMeans, (I) flowPeaks, (J) PAC-MAN, (K) Rclusterpp, (L) SamSPECTRAL, (M) SWIFT, (N) X-shift, (O) immunoClust and (P) k-means. These tools were applied to the same dataset as in Figures 2, 4, 5. Colors are not matched across the tools in this figure. Tools were applied to the full data with 180K cells, except for SWIFT where it was down sampled to 20K cells. Clustering result for Cytometree is not plotted because it generated more than 1,000 clusters. [file Image_2.pdf]

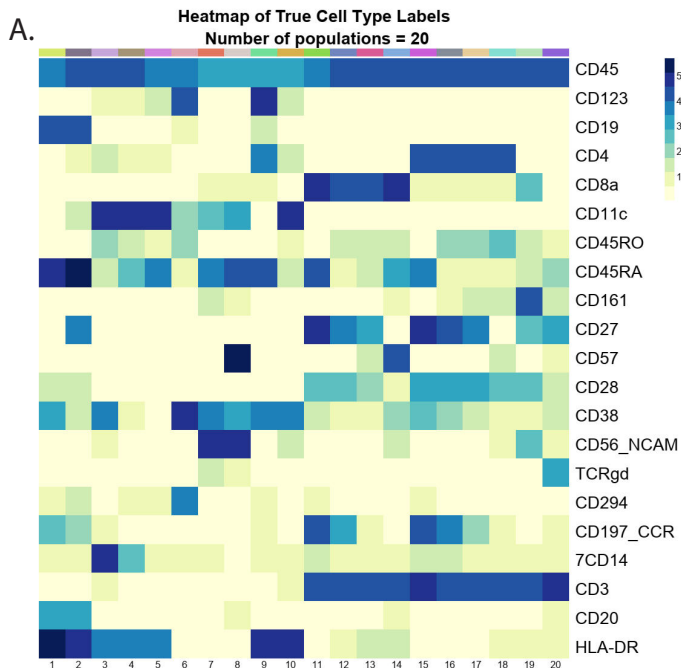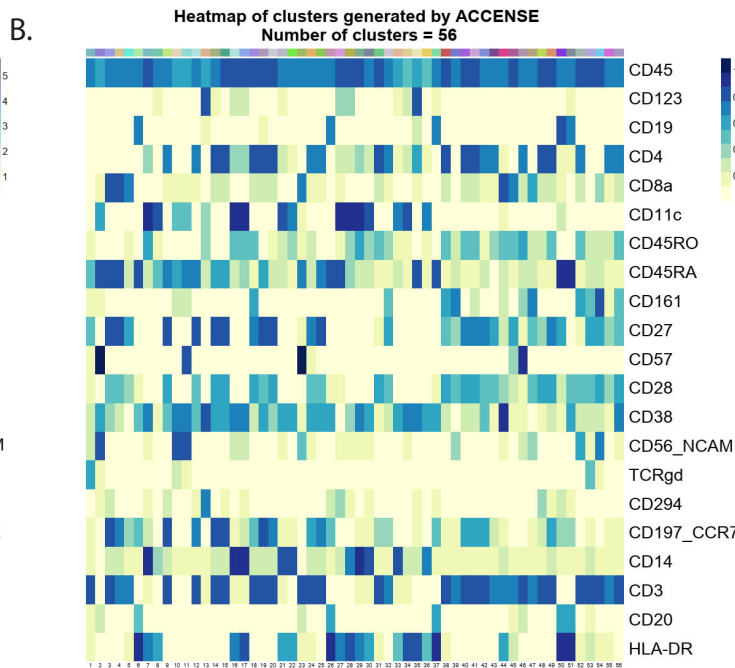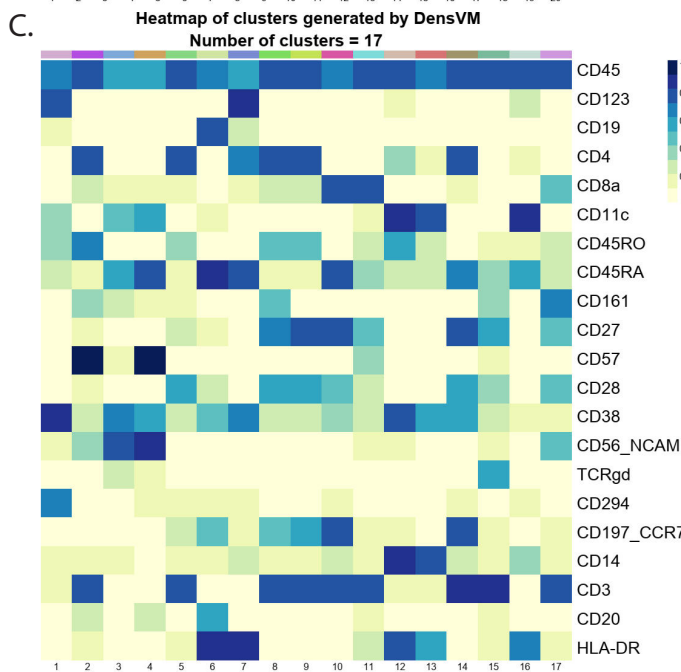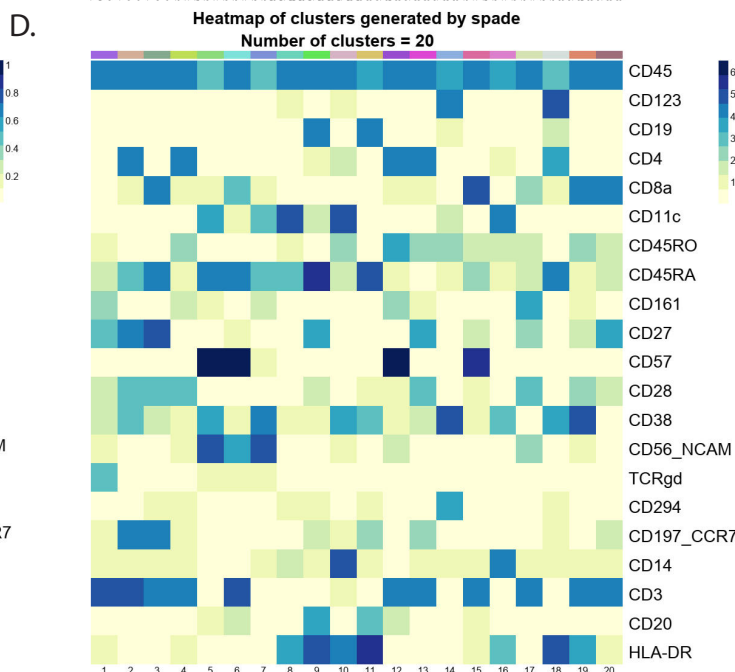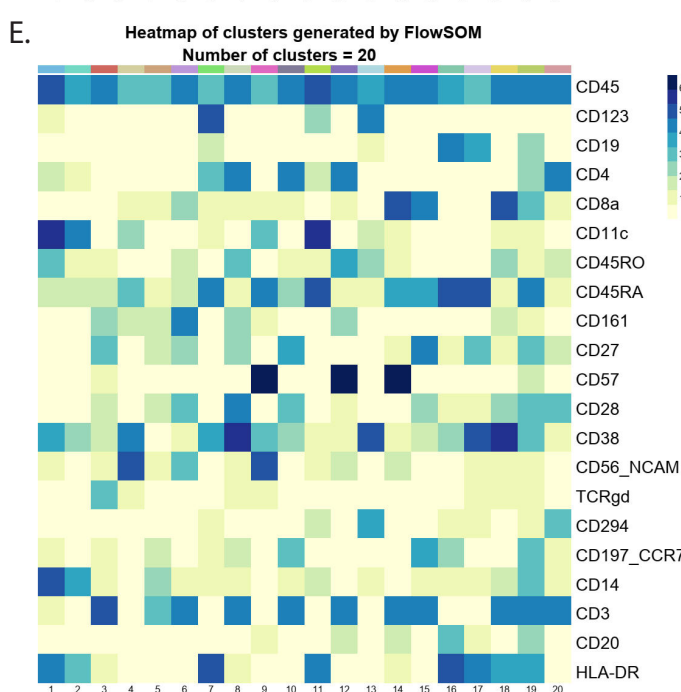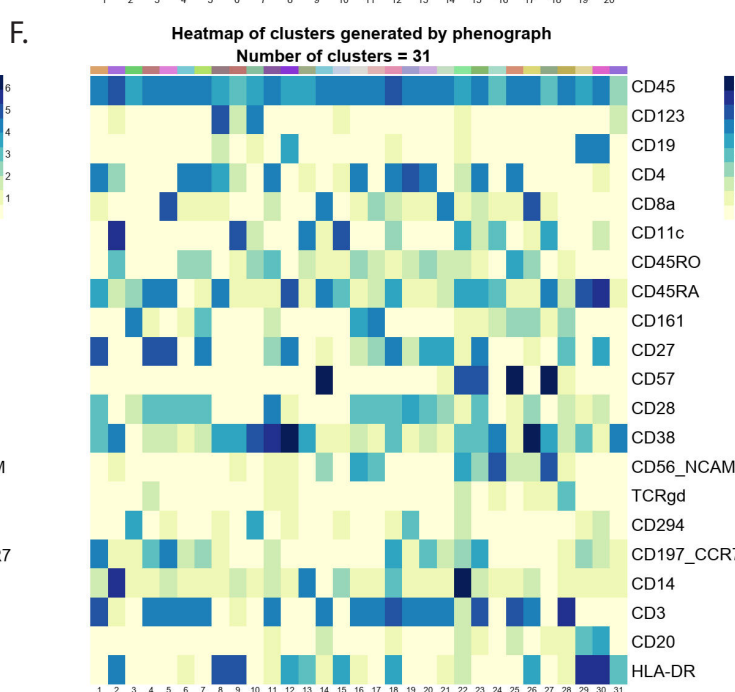

Supplement: FIGURE S3 — Marker expression heatmap for the clustering results Figure 4 and Supplementary Figure S4. (A) Manual gating, (B) ACCENSE, (C) DensVM, (D) SPADE, (E) FlowSOM and (F) PhenoGraph. Manual gating and the five popular tools were applied to a real data to perform cell subpopulation identification. Their results are visualized by heatmap with rows representing markers and columns for clusters. Color darkness level (refer to color bar) indicates the marker median metal intensity across all the cells within a given cluster. [file Image_3.pdf]

A. Manual gating

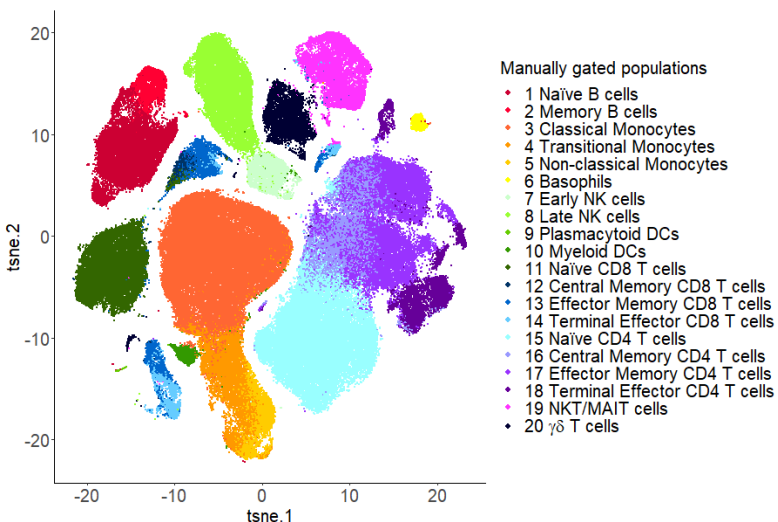

B. ACCENSE

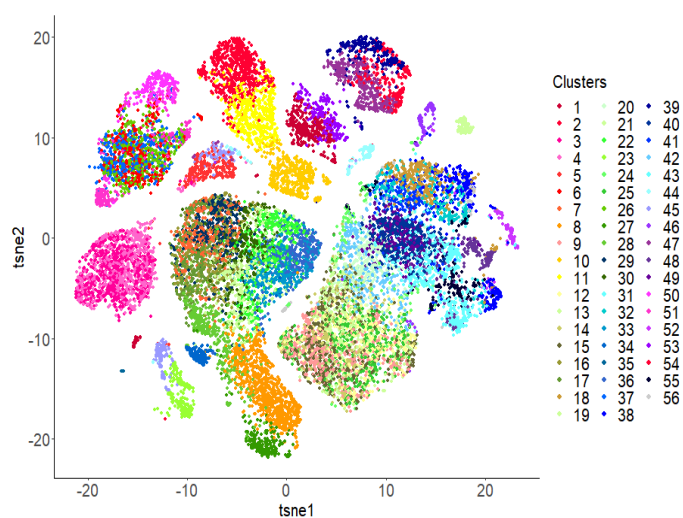

C. DensVM

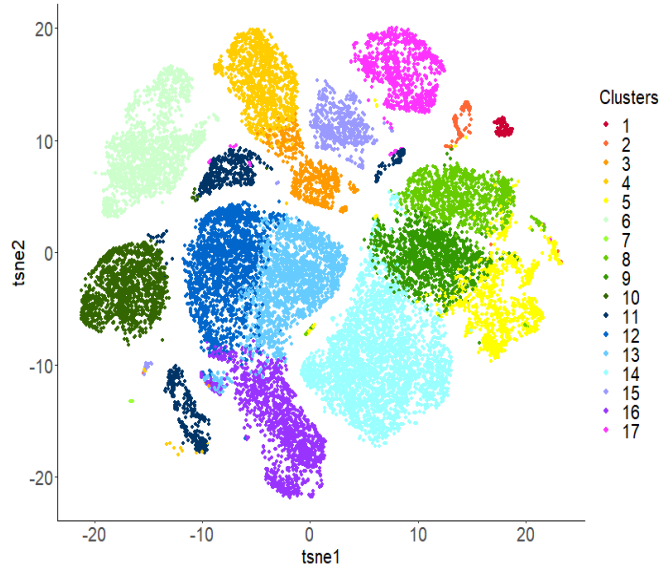

D. SPADE

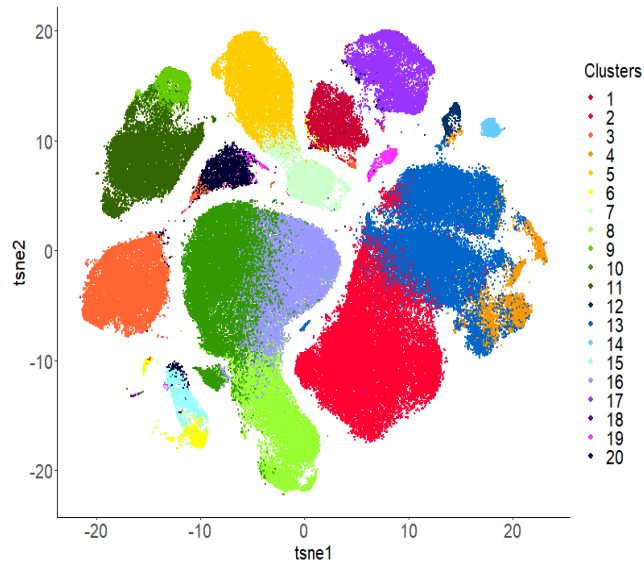

E. FlowSOM

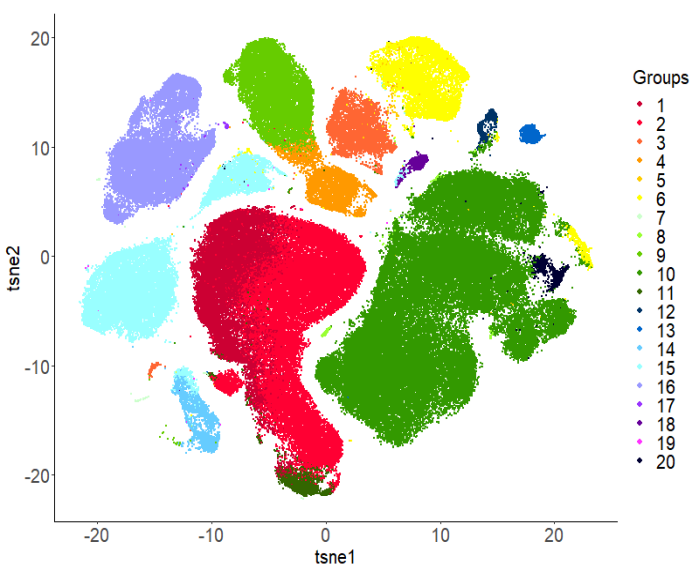

F. PhenoGraph

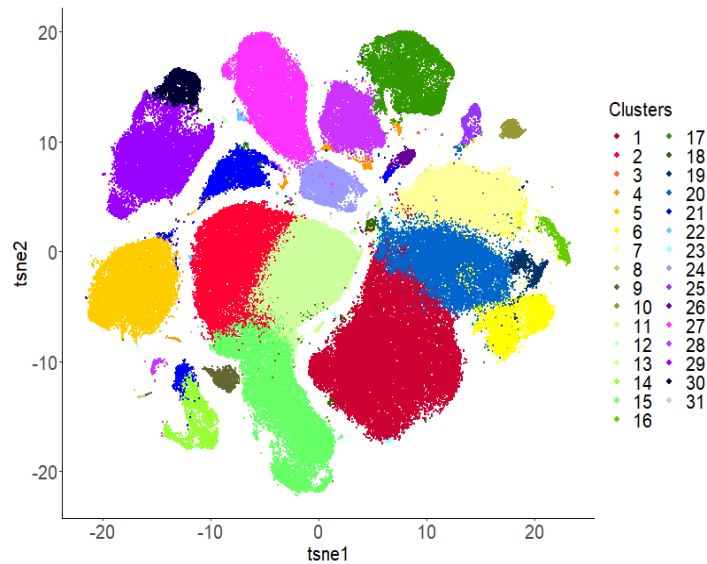

Supplement: FIGURE S4 — t-SNE visualizations for manual gating and five popular unsupervised clustering tools. (A) Manual gating, (B) ACCENSE, (C) DensVM, (D) SPADE, (E) FlowSOM and (F) PhenoGraph. Clusters for each tool are painted with unique colors. Manual gating and the five popular tools were applied to the same data as Figure 2. Tools were applied to the full dataset with 180K cells, except for ACCENSE and DensVM, where the data was down-sampled to 20K cells prior to applying the tools as using the full dataset had a running time greater than 3 h. For SPADE and FlowSOM, we set the number of clusters to 20. For ACCENSE, PhenoGraph and DensVM, the number of clusters was automatically optimized by the tool. [file Image_4.pdf]
